# Global research trends of acupuncture therapy on cancer pain: A bibliometric and visualized study

**DOI:** 10.3389/fonc.2023.1077961

**Published:** 2023-03-06

**Authors:** Chunyu Li, Can Zhao, Jingjing Zhao, Min Wang, Furong Luo, Jianwei Zhou

**Affiliations:** ^1^ Acupuncture and Tuina School/The 3rd Teaching Hospital, Chengdu University of Traditional Chinese Medicine, Chengdu, Sichuan, China; ^2^ College of Pharmacy, Chengdu University of Traditional Chinese Medicine, Chengdu, Sichuan, China; ^3^ Department of Acupuncture, Sichuan Second Hospital of Traditional Chinese Medicine, Chengdu, Sichuan, China; ^4^ Department of Traditional Chinese Medicine, Xi’an No. 3 Hospital, the Affiliated Hospital of Northwest University, Xi’an, Shanxi, China; ^5^ Sichuan Academy of Chinese Medicine Sciences, Chengdu, Sichuan, China

**Keywords:** acupuncture, cancer pain, bibliometric analysis, VOSviewer, web of science

## Abstract

**Background:**

The number of publications on acupuncture for cancer pain is increasing rapidly with an upward tendency. Considering that no bibliometric articles related to this topic have been published yet. It is necessary to evaluate the global scientific output of research in this field, and shed light on the direction of clinical cancer pain management in the future.

**Methods:**

Research publications regarding acupuncture on cancer pain from inception to 2022 were downloaded from the Web of Science Core Collection. Bibliometric analyses were performed using CiteSpace software, the bibliometrix R package, and VOSviewer software. Network maps were generated to assess the collaborations between different countries, institutions, authors, and keywords. And clusters map was generated to evaluate reference.

**Results:**

A total of 790 articles related to acupuncture therapy for cancer pain were identified. We observe that the number of publications is gradually increasing over time. China and the United States were the main contributors. Mem Sloan Kettering Canc Ctr (38 papers) and Beijing Univ Chinese Med (28 papers) contributed the most publications, becoming the leading contributors in this field. Although J Clin Oncol (28 articles) ranked ninth in terms of publication volume, it was the journal with the most citations and the highest number of IF (50.717) and H-index (494) at the same time. MAO J from Mem Sloan Kettering Canc Ctr was the most prolific author (23 articles). The main hot topics included matters related to acupuncture (239 times), pain (199 times), management (139 times), quality of life (107 times), electroacupuncture (100 times), and breast cancer (82 times).

**Conclusion:**

Our bibliometric analysis provides a comprehensive overview of the development of acupuncture for cancer pain, enabling relevant authors and research teams to identify the current research status in this field. At the same time, acupuncture for breast cancer (BC) pain, aromatase inhibitor-induced arthralgia (AIA), and chemotherapy-induced peripheral neuropathy (CIPN) may soon become prospective focus.

## Introduction

Pain continues to be the most common, burdensome and problematic symptoms encountered by patients with cancer, with an incidence rate of 50.7% (1, 2). Physical, emotional and cognitive functioning can be affected by chronic pain, resulting in a decrease in overall quality of life and an increased risk of mortality. The possible causes of widespread pain in cancer patients are as follows: 1) pain caused by tumor metastasis to bones and organs, 2) musculoskeletal symptoms caused by chemotherapy, 3) pain caused by diagnostic examination and drug treatment, and 4) comorbidities (3–5).

The successful management of pain in cancer patients presents a considerable clinical challenge (6). Analgesics have significant adverse effects which not only cause respiratory depression and constipation, but also lead to addiction and tolerance, further reducing the quality of life (7). The NCCN Clinical Practice Guidelines in Oncology (NCCN Guidelines) released in 2019 paid special attention to the theme of cancer pain management, which integrated non-drug methods (8).

Acupoints are sensors of body information. Acupuncture is well-tolerated with little risk of serious side effects. Acupuncture has been shown to have analgesic effects in studies of many other pain diseases, such as knee osteoarthritis, migraine, dysmenorrhea and low back pain (9–12). Due to the multimorphism of cancer pain, multiple investigators have already reported that acupuncture, as an integrative or complementary therapy, can provide effective therapeutic advantages to alleviate cancer-related pain, whether it is acute or chronic pain (13–17). Therefore, despite the positive effect of current therapeutic strategies in improving the survival time, necessary precautions should be adopted along with other treatments to manage cancer pain in the clinical setting (18).

In the current study, we retrieved relevant literature on acupuncture for cancer pain to conduct a statistical analysis by utilizing CiteSpace and VOSviewer. Our study aimed to shed light on the direction of cancer pain management research *via* acupuncture and provide inspiration for researchers to cooperate in their future studies.

## Research methodology

### Sources of data and search strategy

This study collects bibliometric data on acupuncture and cancer pain research for its review. To avoid omissions, the authors conducted the synonyms for “cancer” and “acupuncture” through the MeSH Database in PubMed. All data were collected from the online database Science Citation Index-Expanded (SCI-E) of the Web of Science (WOS). The search time was from database inception to 12 October 2022. The language was restricted to English. There were no restrictions in terms of document type, data category, or document year. The specific search strategy and results are shown in **Table 1**. There were 841 original records in total, including articles, editorial materials, letters, meeting abstracts, and reviews. Finally, we imported these articles into CiteSpace for de-duplication, which removed 51 documents, and thus, 790 results were retained.

**Table 1:** The Topic Search Query.

Set	Results	Search Query
#1	24,575	TS=(Acupunture)) OR TS=(Electroacupuncture))
		OR TS=("electro-acupuncture")) OR TS=(Acupressure)) OR
		TS=(Moxibustion)) OR TS=("Acupoint Injection")) OR
		TS=(Acupoints)) OR TS=(Pharmacoacupuncture)) OR
		TS=("Needle knife")) OR TS=("catgut embedding")) OR
		TS=("catgut implantation at acupoint")) OR
		TS=("embedding thread")
#2	4,297,341	(TS=(tumor*)) OR TS=(tumour*)) OR TS=(cancer*))
		OR TS=(carcin*)) OR TS=(oncolog*)) OR TS=(neoplas*))
		OR TS=(malignan*)
#3	614,101	TS=(pain)
#4	841	#1 AND #2 AND #3

### Assessing

To evaluate the final corpus of 790 articles related to acupuncture for cancer pain, this study adopts a bibliometric analysis approach for its review. Bibliometrics on acupuncture and cancer pain were visualized by using CiteSpace (Version 6.1 R3), R software (version 4.2.1), the bibliometrix R package, VOSviewer (Version 1.6.18), and Microsoft Excel 2019. Two researchers independently completed the literature selection, data extraction, and analysis to ensure the reliability of the results. CiteSpace was used for co-authorship network of countries, institutions, authors, cited journals, and references. In order to have a more comprehensive understanding of topics and research frontiers in this field, we used the bibliometrix R package and VOSviewer to analyze keywords at the same time.

The parameters of CiteSpace were set as follows: time slices were 1985-2022, the number of years in each slice was 1, the term source was selected for all selections, and Pruning was Pathfinder and Pruning sliced Networks.

## Results

### Publication output and temporal trend

**Figure 1:**
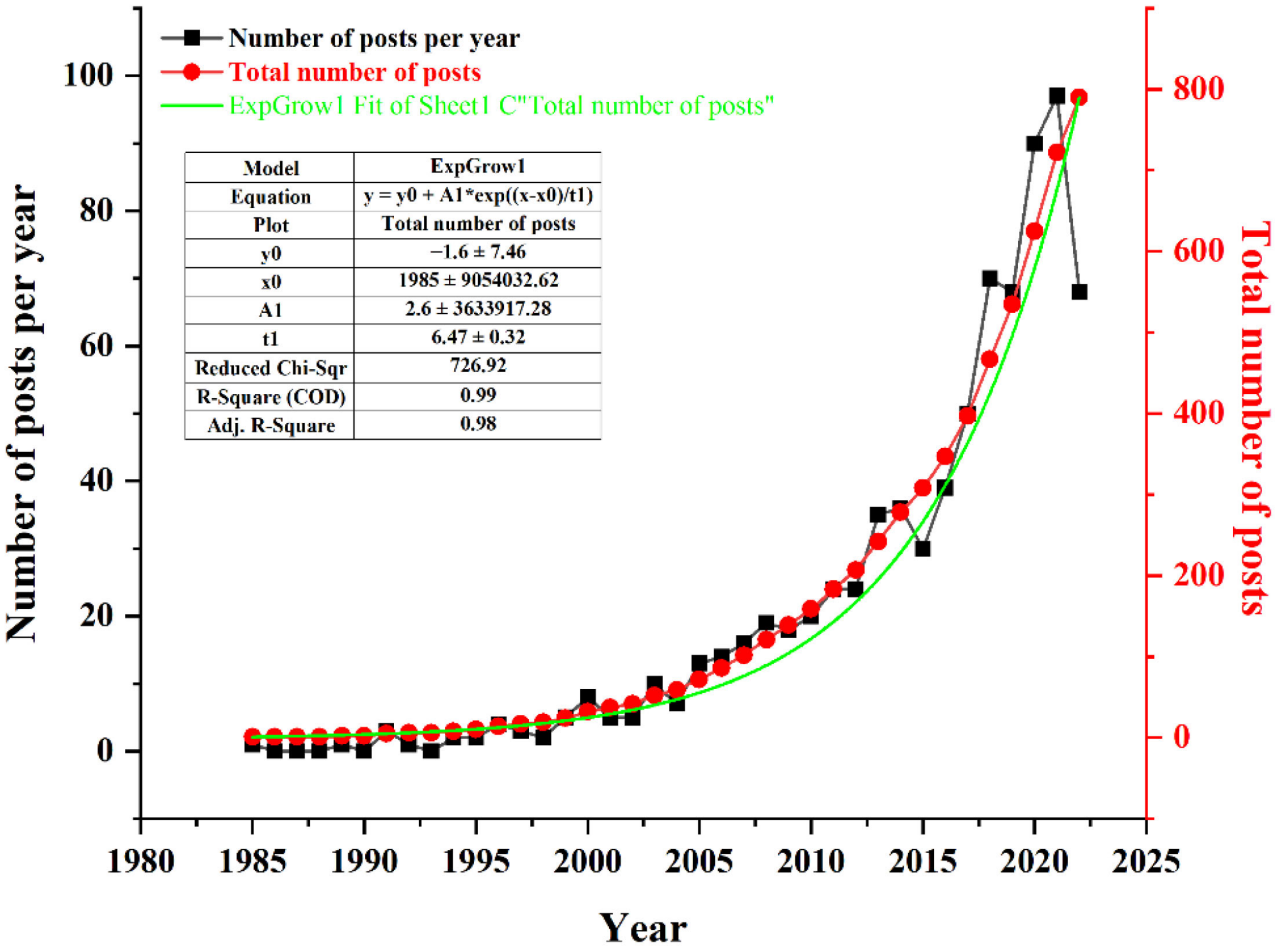
Annual publications covering research on acupuncture for cancer pain.

Based on the above search methods and data processing, a total of 790 publications were obtained, which were published from 1985 to 2022. There were 584 articles (73.92%) and 208 reviews (26.33%) among the 790 included documents, averaging 21 publications per year. The annual distribution of the number of publications is shown in **Figure 1**, showing an increasing growth trend. Before 2005, the annual number of research articles on acupuncture for cancer pain was less than 10 documents. Since then, the publications of related literature on acupuncture and cancer pain showed a small fluctuating upward trend, reaching three peaks in 2014, 2018, and 2021. And 2021 (97 publications, 12.28%) was the most prolific year for publications. To ensure whether the growth of publications on studies of acupuncture for cancer pain conformed to Price’s law, the acquired data were exponentially adjusted and linearly fitted. We obtain the equation y = -1.6+2.6e^((x-1985)/6.47)^(R² = 0.98)from its exponential curve, with the result of good fitting. As a result, the continuous increase in publications over time indicates that the cancer pain aspect of acupuncture therapy is attracting increasing attention, which can provide recommendations for future research.

### Leading countries

**Figure 2:**
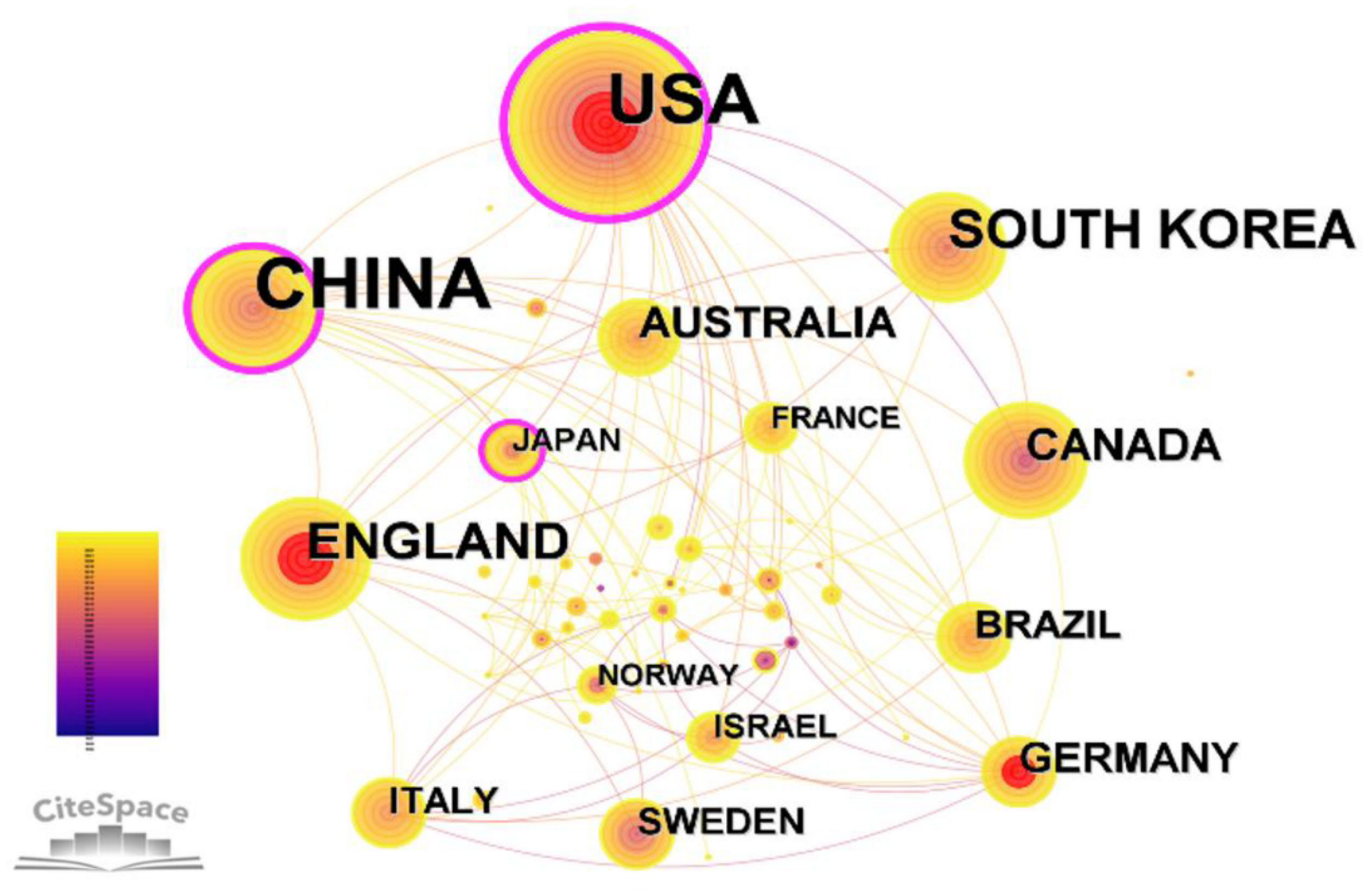
Collaborative map of countries related to the research of acupuncture for cancer pain.

A country collaboration network map was generated by CiteSpace (**Figure 2**). The top 5 most productive countries are presented in **Table 2**. This research involved a set of 54 countries with 118 links. The countries with the most publications were mainly China and the United States. China contributed the highest number of articles (288, 36.46% of all articles), followed by the United States (281, 35.57%), South Korea (67, 8.49%), and England (53, 6.71%). The top five countries by centrality were the United States (0.42), China (0.26), Japan (0.15), Italy (0.09) and Saudi Arabia (0.09). As seen from it, the United States and China have published a large amount of relevant literatures and established collaborative relationships with many countries. Meanwhile, the United States also showed the highest centrality.

**Table 2:** Top 5 Countries with the highest frequency and centrality related to the research of acupuncture for cancer pain.

Ranking	Frequency	Country	Ranking	Centrality	Country
1	288	China	1	0.42	USA
2	281	USA	2	0.26	China
3	67	South Korea	3	0.15	Japan
4	53	England	4	0.09	Italy
5	30	Canada	5	0.09	Saudi Arabia

### Institutions

**Figure 3:**
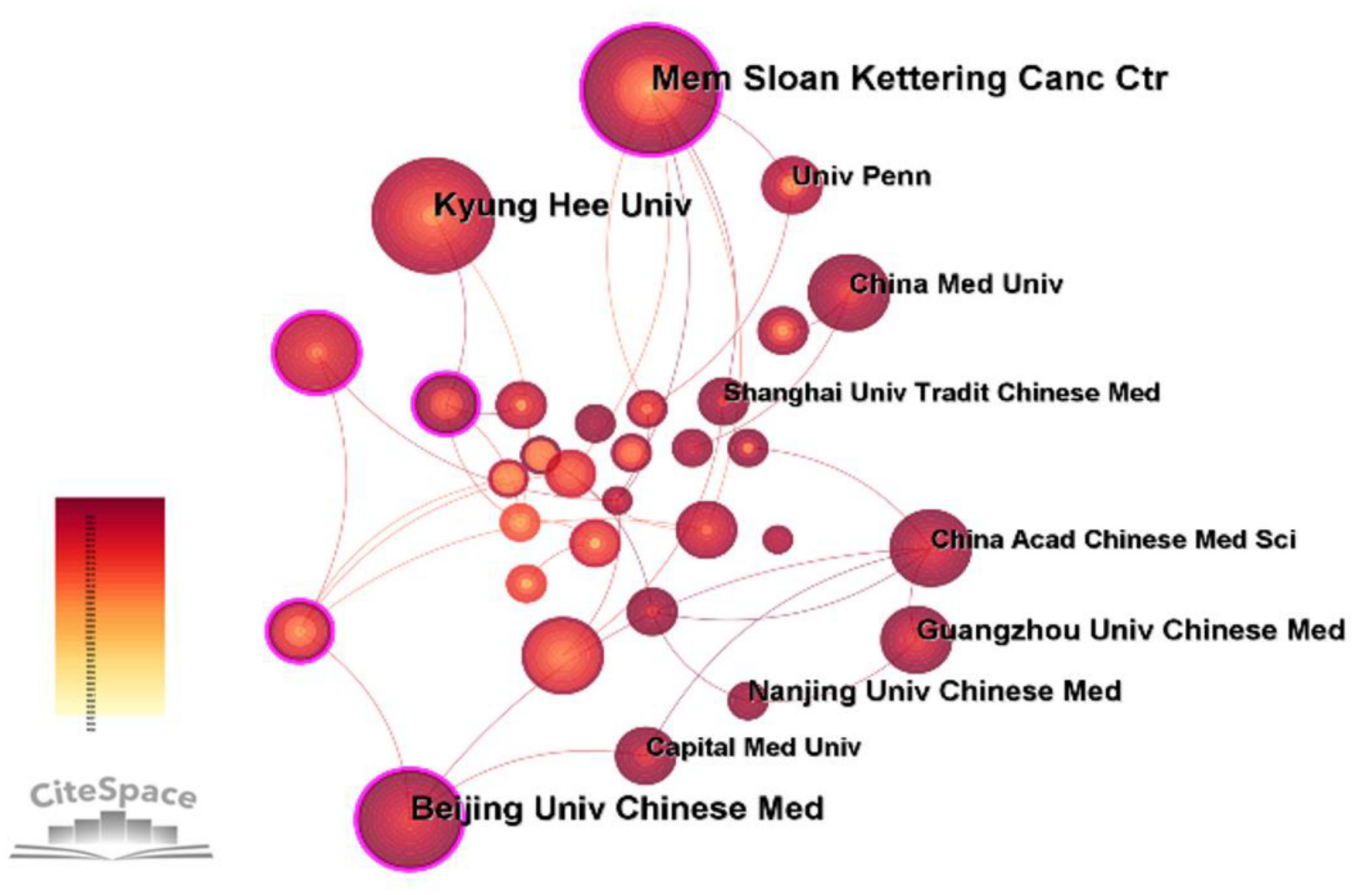
Collaborative map of institutions related to the research of acupuncture for cancer pain.

According to the CiteSpace, a total of 458 institutions were involved in this field (**Figure 3**). The 10 institutions with the highest number of articles were obtained (**Table 3**). Mem Sloan Kettering Canc Ctr (38 records, 4.81% of all articles) contributed the most publications, followed by Beijing Univ Chinese Med (28, 3.54%), Kyung Hee Univ (25, 3.16%), Guangzhou Univ Chinese Med (18, 2.28%), and Nanjing Univ Chinese Med (17, 2.15%). Beijing Univ Chinese Med showed the highest centrality (0.19). Thus, we can see institutions with the most publications and high centrality are mainly distributed in China and the the United States. The purple circle around the nodes reflects the centrality of the network, indicating that Mem Sloan Kettering Canc Ctr and Beijing Univ Chinese Med played a pivotal role in the cooperative relationships among institutions.

**Table 3:** Top 10 institutions related to the research of acupuncture for cancer pain.

Ranking	Frequency	Institution	Country	Centrality	Institution	Country
1	38	Mem Sloan Kettering Canc Ctr	USA	0.19	Beijing Univ Chinese Med	China
2	28	Beijing Univ Chinese Med	China	0.17	Columbia Univ	Canada
3	25	Kyung Hee Univ	South Korea	0.16	Univ Maryland	USA
4	18	Guangzhou Univ Chinese Med	China	0.13	Mem Sloan Kettering Canc Ctr	USA
5	17	Nanjing Univ Chinese Med	China	0.12	Korea Inst Oriental Med	South Korea
6	15	China Med Univ	China	0.09	Dana Farber Cane Inst	USA
7	15	Univ Penn	USA	0.08	Harvard Univ	USA
8	14	Capital Med Univ	China	0.08	Natl Cheng Kung Univ	China
9	14	Shanghai Univ Tradit Chinese Med	China	0.08	British Acupuncture Council	England
10	13	China Acad Chinese Med Sci	China	0.07	Kyung Hee Univ	South Korea

### Authors and cited authors

**Figure 4:**
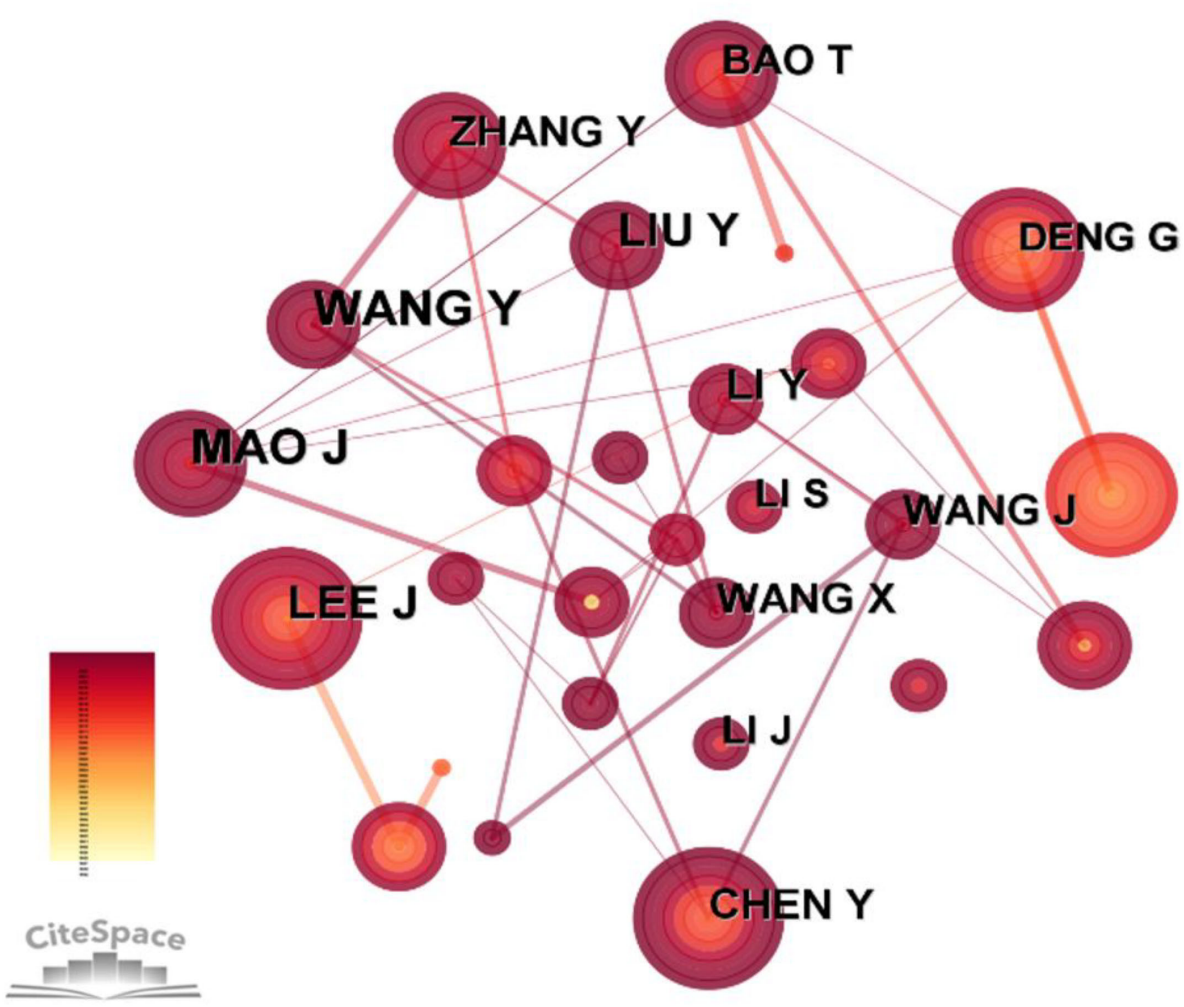
Collaborative map of co-author related to the research of acupuncture for cancer pain.

The CiteSpace software was used to generate a co-author map containing 671 nodes and 1233 links (**Table 4**; **Figure 4**). In terms of the number of published papers, MAO J was the most prolific author, with 23 articles (2.91%), followed by WANG Y (22, 2.78%), LEE J (18, 2.28%) and LIU Y (17, 2.15%). LEE J and DENG G showed the highest centrality, each with the centrality of 0.09. By observing the visualization map, we could find the authors with more publications and higher central position tend to cooperate closely with other authors.

**Table 4:** Top 10 authors of studies on acupuncture for cancer pain.

Ranking	Frequency	Author	Centrality	Author
1	23	MAO J	0.09	LEE J
2	22	WANG Y	0.09	DENG G
3	18	LEE J	0.06	CHEN Y
4	17	LIU Y	0.05	MAO J
5	15	LI Y	0.05	ZHANG Y
6	15	ZHANG Y	0.05	BAO T
7	14	WANG J	0.05	LI Q
8	14	WANG X	0.04	LEE M
9	14	CHEN Y	0.03	LI Y
10	13	BAO T	0.03	WANG J

**Figure 5:**
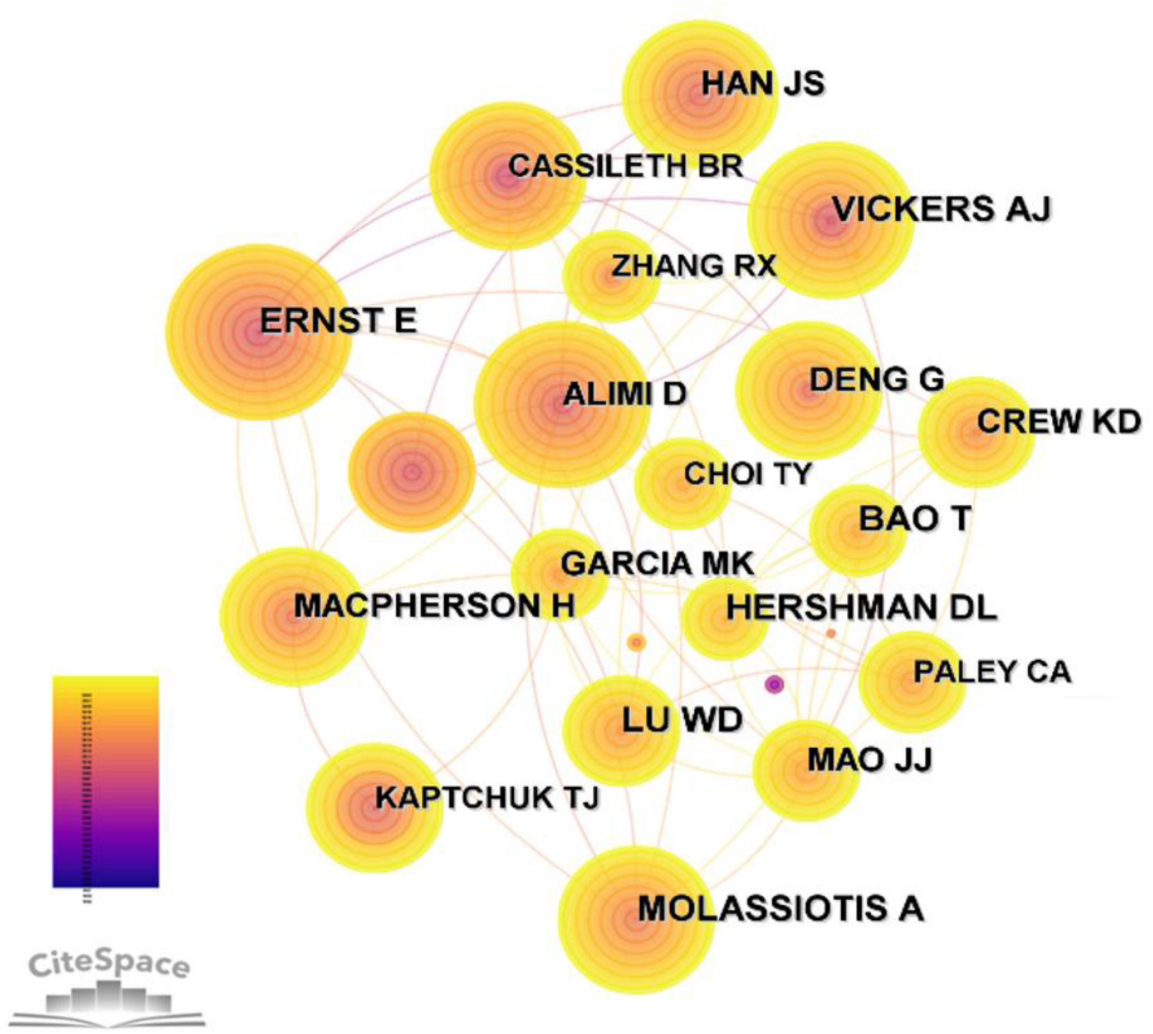
Collaborative map of cited authors related to the research of acupuncture for cancer pain.

An author co-citation map was generated displaying 969 nodes and 3444 links (**Table 5**; **Figure 5**). The top 5 most cited authors were Molassiotis A (102), Vickers AJ (101), Ernst E (93), Lu WD (93), and Hershman DL (92). The top 5 authors in centrality were Cassileth BR (0.2), Ernst E (0.19), Vickers AJ (0.1), Molassiotis A (0.09), and, Shen JN (0.09).

**Table 5:** Top 5 Cited authors of studies on acupuncture for cancer pain.

Ranking	Cocitation counts	Cited author	Centrality	Cited author
1	102	Molassiotis A	0.2	Cassileth BR
2	101	Vickers AJ	0.19	Ernst E
3	93	Ernst E	0.1	Vickers AJ
4	93	Lu WD	0.09	Molassiotis A
5	92	Hershman DL	0.09	Shen JN

### Leading journals and cited journals

In total, 297 academic journals published papers about acupuncture for cancer pain. **Table 6** lists the top 10 most popular journals contributing to articles on acupuncture and cancer pain topics, and shows the countries of origin and the impact factor of the top 10 journals. The top 10 journals published about 34.43% of the documents in the field. Among them, the average impact factor (IF) was 7.5572. Evid-Based Compl Alt was the leading journal, publishing the most papers (42 articles, England), followed by Integr Cancer Ther (40 articles, United States), Medicine (33 articles, United States), Supportive Care In Cancer (30 articles, United States), J Altern Complem Med (30 articles, United States), J Pain Symptom Manag (28 articles, United States), Acupuncture In Medicine (26 articles, England), Complement Ther Med (15 articles, England), J Clin Oncol (14 articles, United States), and Acupuncture Electro (14 articles, United States). The impact factor (IF) of Evid-Based Compl Alt (the most published journal) was 2.650 (2021), and the H-index was 72. Among the top 10 journals, the journal with the highest IF (50.717), which also had the highest H-index (494), was J Clin Oncol from the United States. It indicates that the articles published in this journal are influential in the field of acupuncture for cancer pain.

**Table 6:** Top 10 journals related to studies on acupuncture for cancer pain.

Ranking	Articles	Journal	IF(2021)	H-index	Region
1	42	Evid-Based Compl Alt	2.650	72	England
2	40	Integri Cancer Ther	3.077	53	USA
3	33	Medicine	1.817	135	USA
4	30	Supportive Care in Cancer	3.359	98	USA
5	30	J Altern Complem Med	2.381	80	USA
6	28	J Pain Symptom Manag	5.576	129	USA
7	26	Acupuncture in Medicine	1.976	42	England
8	15	Complement Ther Med	3.335	55	England
9	14	J Clin Oncol	50.717	494	USA
10	14	Acupuncture Electro	0.684	24	USA

**Figure 6:**
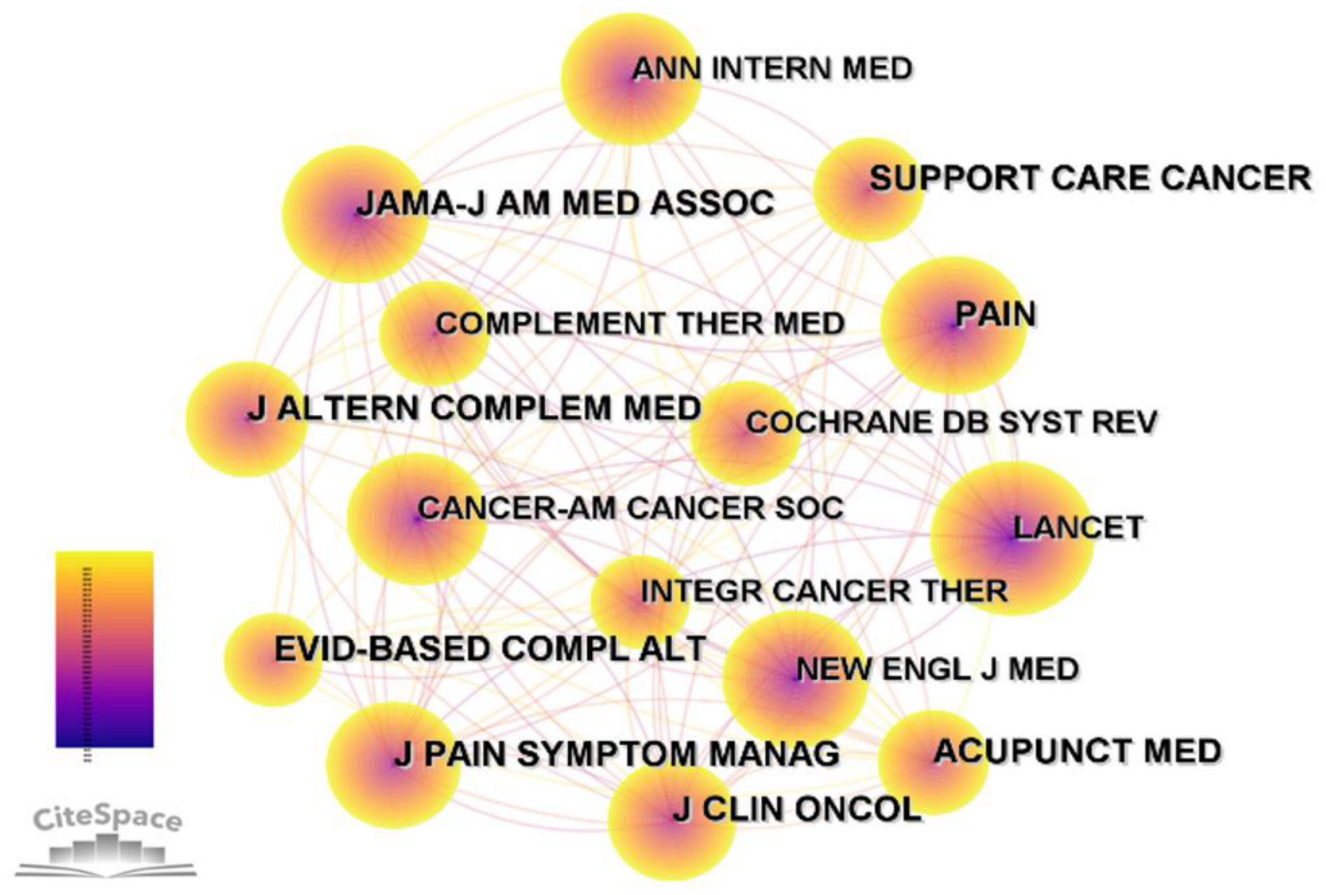
Co-citation map of journals related to the research of acupuncture for cancer pain.

**Table 7** and **Figure 6** present the top 5 cited journals on acupuncture for cancer pain research. J Clin Oncol was cited in the most journals (391 counts), followed by Pain (360 counts). The highest citation counts of this journal may be due to it being the journal with the highest IF and the highest H-index. This also provides a direction for us to find related articles in the future. Evid-Based Compl Alt ranked third, with 324 counts. The fourth and fifth, with more than 250 citations, were Acupunct MED (285 counts) and Support Care Cancer (275 counts), respectively. A node represents a journal, the purple ring outside the node indicates the size of the centrality of the journal. Brit MED J has the highest centrality (0.15).

**Table 7:** Top 5 cited journals with the highest frequency and centrality related to studies on acupuncture for cancer pain.

Ranking	Cocitation counts	Cited journal	Centrality	Cited journal
1	391	J Clin Oncol	0.15	Brit MED J
2	360	Pain	0.11	Am J Chinese Med
3	324	Evid-Based Compl Alt	0.08	Anesth Analg
4	285	Acupunct MED	0.08	Am J Med
5	275	Support Care Cancer	0.07	Ann Intern Med

### Reference

**Figure 7:**
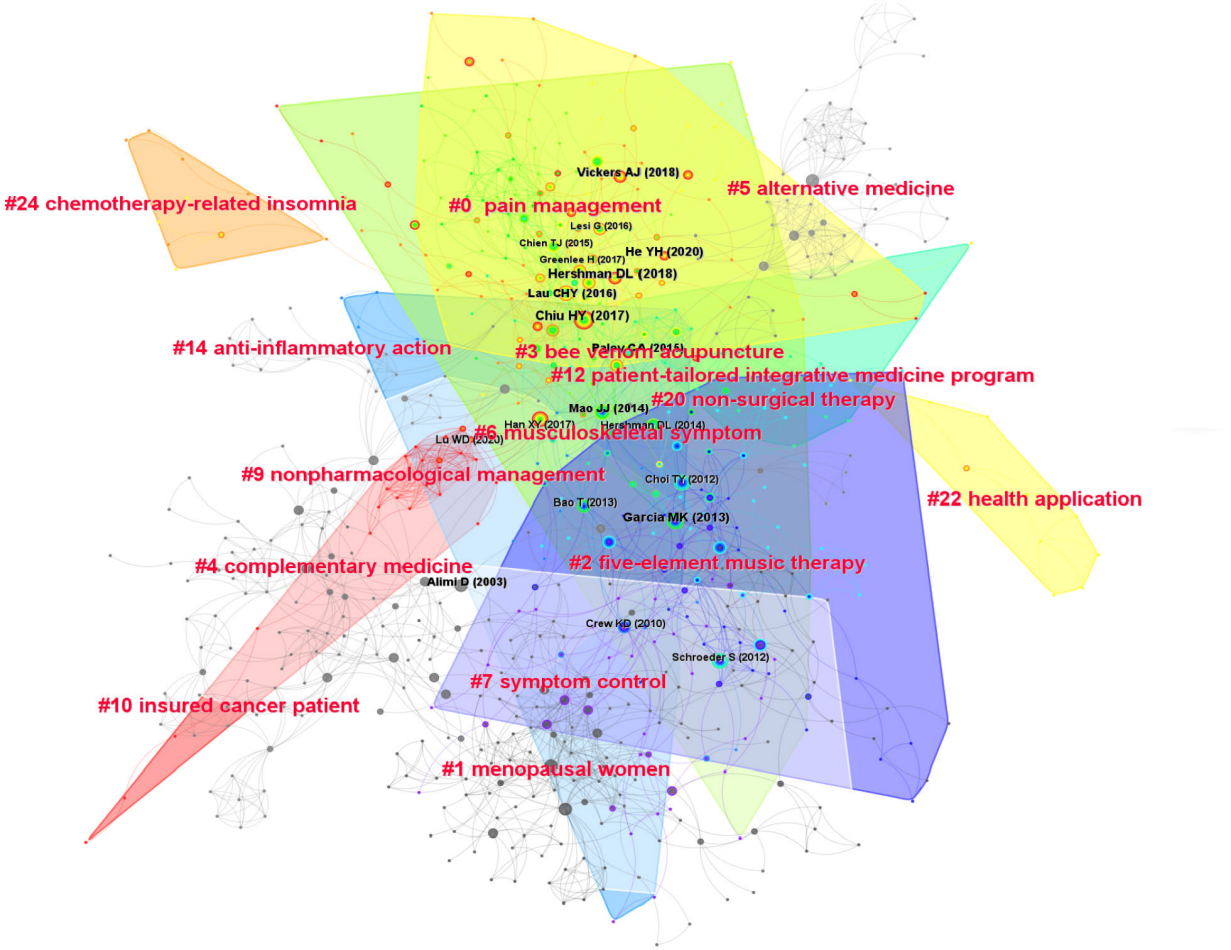
Cited references map related to the research of acupuncture for cancer pain.

We used CiteSpace to obtain a reference co-citation map related to studies on acupuncture for cancer pain (**Figure 7**), with 916 nodes and 2522 links. **Table 8** enumerates the basic information of the top 10 most cited references. These highly cited studies were mainly published between 2013 and 2020. The most cited reference was Hershman DL’s paper, which was a randomized clinical trial (RCT) of the acupuncture effects on joint pain related to aromatase inhibitors among women with early-stage breast cancer, published in JAMA in 2018. It was cited 41 times. Chiu HY (2017) wrote the second most highly cited paper, with 37 citations. He YH (2020) wrote a systematic review, ranked third, with 33 citations. The fourth position was occupied by Garcia MK (2013), with 29 citations. Followed by Mao JJ (2014), Paley CA (2015), and Vickers AJ (2018), with 23 citations. A total of 24 clusters were obtained. The largest cluster was “pain management” which contains 104 references. The color of the cluster “complementary medicine”, “nonpharmacological management”, and “insured cancer patient” were red, indicating the latest research direction of research on acupuncture in treating cancer pain.

**Table 8:** Top 10 most frequently cited references related to studies on acupuncture for cancer pain.

Ranking	Frequency	Title	Author and Publication Year	DOI
1	41	Effect of acupuncture vs sham acupuncture or waitlist control on joint pain related to aromatase inhibitors among women with early-stage breast cancer: a randomized clinical trial	Hershman DL (2018)	10.1001/jama.2018.8907
2	37	Systematic review and meta-analysis of acupuncture to reduce cancer-related pain	Chiu HY (2017)	10.1111/ecc.12457
3	33	Clinical evidence for association of acupuncture and acupressure with improved cancer pain: a systematic review and meta- analysis	He YH (2020)	10.1001/jamaoncol.2019.5233
4	29	Systematic review of acupuncture in cancer care: a synthesis of the evidence	Garcia MK (2013)	10.1200/JCO.2012.43.5818
5	23	A randomised trial of electro-acupuncture for arthralgia related to aromatase inhibitor use	Mao JJ (2014)	10.1016/j.ejca.2013.09.022
6	23	Acupuncture for cancer pain in adults	Paley CA (2015)	10.1002/14651858.CD007753.pub3
7	23	Acupuncture for chronic pain: update of an individual patient data meta-analysis	Vickers AJ (2018)	10.1016/j.ipain.2017.11.005
8	22	Acupuncture and related therapies for symptom management in palliative cancer care: systematic review and meta-analysis	Lau CHY (2018)	10.1097/MD.0000000000002901
9	21	Acupuncture and related therapies for symptom management in palliative cancer care: systematic review and meta-analysis	Alimi D (2016)	10.1200/JCO.2003.09.011
10	20	Acupuncture combined with methylcobalamin for the treatment of chemotherapy-induced peripheral neuropathy in patients with multiple myeloma	Han XY (2003)	10.1186/s12885-016-3037-z

### Keywords

**Figure 8:**
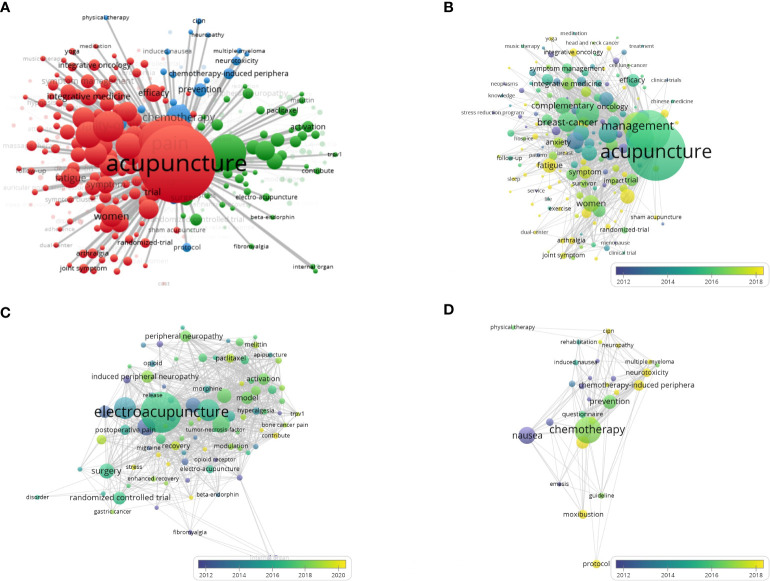
Co-occurrence analysis of keywords of research on acupuncture for cancer pain. **(A)** Mapping of predominant keywords of research. Footnote: The keywords were classified into 3 clusters, presented by three colors (red, green, and blue). **(B–D)** Overlay visualization of red cluster keywords, green cluster keywords, and blue cluster keywords by time (blue: earlier, yellow: later).

Co-occurrence analysis of keywords can identify research hotspots and trends. We analyzed a total of 90 keywords among 2937 keywords related to the research of acupuncture for cancer pain that were identified as having occurred more than fifteen times. The top 90 keywords are visualized in **Figure 8A**, showing that predominant words were divided into three clusters, represented by three colors (red, green, and blue).

**Figure 9:**
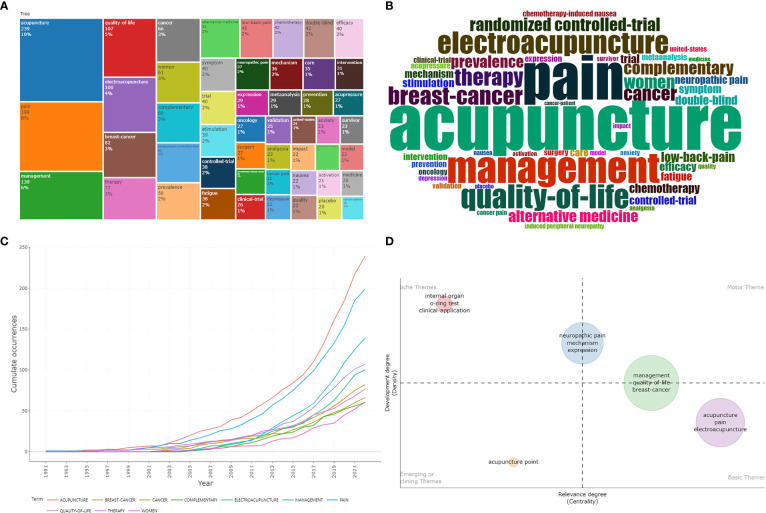
**(A, B)** Tree map and word cloud map of keywords. **(C)** Growth trends of the top 10 keywords in the research of acupuncture for cancer pain. **(D)** Thematic Map of keywords of studies.

We can see a visualization of each cluster of keywords over time (**Figures 8B–D**). Cluster 1 refers to acupuncture therapy method for cancer pain, indicated by red, with the main keywords of acupuncture, management and breast cancer. Cluster 2 refers to electroacupuncture therapy method for cancer pain, indicated by green, with the main keywords of electroacupuncture, induced peripheral neuropathy and postoperative pain. Cluster 3 refers to disease, indicated by blue, with the main keywords of chemotherapy, chemotherapy induced periphera, neurotoxicity, and multiple myeloma, which were the latest topics. The development trend and strategic coordinate map are shown in **Figure 9**. The high-occurrence words (**Figures 9A, B**) include acupuncture (239 times), pain (199 times), management (139 times), quality of life (107 times), electroacupuncture (100 times), breast cancer (82 times), therapy (77 times), cancer (66 times), women (61 times), complementary (60 times). It can be seen that the covered groups with the need to treat cancer pain by acupuncture mainly existed in women, and their negative emotions were mainly anxiety and depression. Acupuncture was most likely to be considered as an alternative therapy to provide effective palliative treatment for cancer pain patients, and improve their quality of life. The cancer pain categories treated by acupuncture were mainly breast pain, neuropathic pain, and low back pain, which were characterized by chronic pain. In addition, we can also see that these treatments were mostly achieved through RCTs. **Figure 9C** shows that the frequency of the above top 10 keywords increased over time. The keyword “acupuncture” grew the fastest, followed by “breast cancer”. Through the thematic map of keywords (**Figure 9D**), internal organ, o-ring test, and clinical application were highly developed and isolated themes; acupuncture point wes emerging or declining themes; acupuncture, pain; electroacupuncture were basic and transversal themes. In addition, we can predict that neuropathic pain, mechanism, expression, management, quality of life, and breast cancer will be the research trends.

## Discussion

In the present research, we performed a bibliometric analyses using VOSviewer, CiteSpace, and the bibliometrix R package to characterize the current landscape and frontier topic of acupuncture for cancer pain. The contributions of countries, institutions, authors, journals, reference and keywords to this emerging field were analyzed. Thus, the general information was summarized, predicting hotspots and trends on acupuncture for cancer pain.

### Basic information

Since 2005, the annual publication output in this field has increased in a steady fashion. The publication output in 2021 was the highest, accounting for 12.28% of all the included studies. It can be seen that acupuncture on cancer pain has attracted more and more attention, and various countries have begun to using acupuncture to treat patients with cancer pain. The reason why acupuncture attracts more and more attention to this field is that, on the one hand, the development of the modern medicine is more and more mature and the patients’ quality of life is concerned. On the other hand, the reason may be the widely use of acupuncture in medical fields has been proved to be effective.

China was the country with the largest number of publications, but the United States had a high centrality of 0.42, indicating that most countries in this field had direct and indirect cooperation with the United States. The institutions with the most publications were in the United States. And the institution with the largest number of publications in China is Beijing Univ Chinese Med. It may be concluded that Chinese researchers, with the advantage of the long history use of acupuncture, are equipped to conduct more studies and publish more literature, while western researchers are more influential in the study of cancer pain. According to the analysis of publishing institutions, the first and second institution were Mem Sloan Kettering Canc Ctr and Beijing Univ Chinese Med respectively, both of which have very strong comprehensive strengths. Mem Sloan Kettering Canc Ctr is the largest private cancer research center in the world, making a significant contribution to the understanding, diagnosis and treatment of cancer. Beijing Univ Chinese Med is the most important medical innovation research base in China, training a large number of senior medical and health personnel. These results suggest that the two institutions mentioned above, may significantly influence the direction of studies in this field and participate in the strongest cooperation globally.

Although J Clin Oncol ranked ninth with 28 published articles, it had the most citations with the highest IF (50.717) and H-index (494) at the same time, indicating that this journal is very influential in this field, which can provide inspiration for future research. MAO J, a doctor from Mem Sloan Kettering Canc Ctr, who had the largest number of publications (23, 2.91%), is a pioneer in the field of acupuncture in the treatment of cancer pain. With the increase of cancer incidence and mortality, MAO J believed that the current cancer care had been challenged. Therefore, he proposed an integrative medicine (TCIM) including acupuncture and massage, to alleviate cancer symptoms or treatment-related adverse reactions such as pain, insomnia and fatigue (19). The author with the highest cited frequency is Molassiotis A, whose most cited paper is a RCT published in Support Care Cancer in 2002, which focused on adjuvant intervention to improve the standards of care for cancer patients with side effects of chemotherapy (20).

### Research frontiers and trends

Keywords and references reflect the content of the research, which is helpful to identify hotspots and frontiers from their frequency, centrality, and clustering distribution.

Based on the co-occurrence map of keywords and references map, it can be determined that acupuncture and electroacupuncture (EA) may be the main therapeutic therapies for cancer pain. In addition, the management of cancer pain probably have received the greatest concern from researchers in this field. We can also infer that studies on the treatment of cancer pain with acupuncture have covered a variety of types, including breast pain, arthralgia, neuropathic pain, and low back pain.

#### Breast cancer pain

According to the global cancer statistics in 2020, female breast cancer (BC) has surpassed lung cancer to become the most commonly diagnosed cancer type and the leading cause of cancer-related deaths among women (21). To our knowledge, breast cancer increases the susceptibility to menopausal symptoms such as joint pain, headache, mood changes, depressive, paresthesia and tingling (22, 23). Post-mastectomy pain syndrome (PMPS) mainly involves the chest, axilla, and ipsilateral upper extremity (24). Chronic post-operative pain is so common among BC patients in part because it can arise for a variety of reasons, including the existence of a preoperative painful condition, axillary lymph node dissection, intercostobrachial nerve damage during surgical dissection, acute postoperative pain, and psychological factors (25, 26).

Acupuncture has demonstrated its effectiveness in managing symptoms of BC survivors (27). Existing systematic reviews have proved that acupuncture can not only relieve pain in patients with BC, but also improve hot flashes, fatigue, sleep disturbance, anxiety, and especially the quality of patients with BC (23, 28, 29). The Society for Integrative Oncology (SIO) developed an evidence-based guideline on the use of integrative therapy during and after breast cancer treatment, which recommends acupressure and acupuncture to manage adverse effects related to breast cancer treatment (30).

#### Aromatase inhibitor-induced arthralgia

Aromatase inhibitors (AIs), as a standard treatment for early-stage breast cancer, have adverse effects including headache and arthralgia, which in turn may cause poor adherence to AIs. Aromatase inhibitor-induced musculoskeletal symptoms (AIMSS) is characterized by symmetric pain or soreness in multiple joints, musculoskeletal pain and morning stiffness (31, 32). Through the analysis of keyword, we can see that RCT was the most common research method with the strongest evidence to prove its effectiveness. RCTs have reported that acupuncture or EA can significantly improve joint pain and stiffness in BC women with aromatase inhibitor-induced arthralgia (AIA) (33–35). Moreover, systematic reviews have demonstrated that acupuncture can significantly reduce pain intensity of breast cancer patients with AIA. what’s more, acupuncture treatment has no significant side effects (36–40).

#### Chemotherapy-induced peripheral neuropathy

Chemotherapy-induced peripheral neuropathy (CIPN) is a common clinical problem in cancer patients, which often leads to acral pain (41). PRICE, S put forward the treatment protocol of acupuncture as an auxiliary nursing care for chemotherapy patients as early as 2006 (42). Of concern, Rostock M and Greenlee, H respectively adopted EA protocols for cancer patients receiving chemotherapy, however, did not found a positive effect on the prevention and treatment of CIPN (43, 44). Later, BAO, T showed the efficacy and safety of acupuncture in reducing the incidence of high-grade CIPN during chemotherapy in a single-arm phase IIA trial, but he also called for a follow-up randomized controlled trial to establish definitive efficacy (45).

A randomized pilot study published in 2020 revealed that an 8-week acupuncture intervention could improve neuropathic sensory symptoms in breast cancer survivors with mild and moderate CIPN after the completion of taxane-containing adjuvant chemotherapy (43). Then, other RCTs also demonstrated that acupuncture can alleviate the neuropathic pain (eg, hand numbness, tingling, and pain) of CIPN and increase touch perception thresholds (46, 47). In addition, several systematic reviews have been published in 2022 to evaluate the effects of acupuncture therapies on CIPN (48–50). According to the theory of traditional Chinese medicine, the acupoints (eg, Qihai, Neiguan, Hegu, Zusanli, Sanyingjiao) selected to relieved CIPN in cancer patients are characterized by tonifying Qi, regulating Qi and blood circulation, and treating localized symptoms.

#### Analgesic mechanisms

The underlying mechanism of acupuncture applied to cancer pain is still not completely understood. As to acupuncture analgesia, inflammatory pain animal models actually reported that many bioactive chemicals (such as β-endorphins, IL-1β, dynorphines, substance P) were involved in acupuncture inhibition of cancer pain (51). Regarding acupuncture for CIPN, certain studies demonstrated that it may involve stimulation of Aδ and C nerve fibers or α2 and β-adrenoceptors (52). CHOI J W found that EA stimulation of the ST36 acupoint, mediated by spinal opioid receptor, alpha2- and beta-adrenoceptors, significantly reduced paclitaxel-induced neuropathic pain in mice (53). WANG F J believes that EA reduces allodynia mainly by restoring the Nrf2/HO-1 signaling pathway (54).

#### Psychological factors

Psychosomatic symptoms always plague most cancer patients, especially women (55). Those who survive tend to leave long-term chronic pain, often accompanied by negative emotions such as anxiety and depression, which have a significant impact on their quality of life (56). In turn, this will have a negative impact on the comprehensive treatment of cancer patients. A cross-sectional comparative study pointed out that cancer patients with pain features showed greater psychological barriers (57). Recent systematic review indicated that psychological intervention can help reduce cancer-related pain in adults (58). In this way, we can infer that emotional factor play a key role in the management of cancer patients. Acupuncture plays a direct or indirect role in cancer pain management by increasing plasticity in the hippocampus and neural networks, reducing inflammation in the brain, and alleviating negative emotions (59, 60). Therefore, we need to strengthen interprofessional and multidisciplinary treatment and emphasize the importance of psychotherapy in cancer pain management, which requires enhanced doctor-patient communication, social support and patient cognitive behavioral therapy.

#### Other frontiers

Due to the emergence of low-correlation keywords, we may have ignored the latest research trends. Fortunately, we found several new trends. LEE J reported in an RCT that acupuncture could alleviate the pain and dysfunction of cancer patients with a history of neck dissection, and relieve dry mouth. In this study, LI-4, SP-6, GV-20, luozhen, auricular shenman, local ashi tender points and LI-2 were selected (61). Mao J treated cancer survivors with chronic musculoskeletal pain for 10 times with EA or auricular acupuncture, which reduced the average Brief Pain Inventory (BPI) pain severity score by 1.9 points and 1.6 points respectively. And electroacupuncture produced greater pain relief (62).

## Conclusion

To the best of our knowledge, this study is the first bibliometric paper focused on publications related to acupuncture for cancer pain in this field worldwide. More and more attention has been paid to acupuncture treatment of cancer pain. The types of cancer pain are mainly BC pain, AIA, and CIPN. The pathogenesis of acupuncture in the treatment of cancer pain remains unclear, and there is a lack of unified and recognized treatment strategies. Although the efficacy and safety of acupuncture in the treatment of cancer pain have been confirmed and recognized, the use of acupuncture points is not uniform. Acupuncture taken in different ways and evaluation methods are different, and researchers have not reached a consensus. Therefore, how to better focus on the pain status of cancer patients, integrate various auxiliary means of traditional Chinese medicine, standardize clinical operations, and care for patients’ psychological health will be the direction of future research.

In conclusion, this study summarizes the data of published research papers and provides a bibliometric reference for further research in the field of cancer pain. However, the treatment of cancer pain with acupuncture still needs further exploration by scholars. Whether it is intervention mechanism, comprehensive pain assessment, management of pain crisis, or continuous care for cancer pain, it needs to be discussed and advanced. Moreover, cooperation among countries, regions, authors and disciplines can be strengthened, and more research can be done on acupuncture for cancer pain.

## Limitations

There are some limitations in this study. Firstly, our retrieval time is from database inception to October 12, 2022. On the one hand, the documents before 1985 are not included in the database, and on the other hand, the database is constantly updated, which will lead to incomplete literature retrieval. Secondly, we try to use as many terms as possible as search terms, there may still be a lot of relevant terms of research may be left out, which may lead to the neglect of the latest research trends.

## Data availability statement

The raw data supporting the conclusions of this article will be made available by the authors, without undue reservation.

## Author contributions

CYL contributed to conception and design of the study. CYL and CZ collected and analyzed experimental data. CZ, MW, FRL and JJZ performed the statistical analysis. CYL wrote the paper. JWZ revised the paper for intellectual content. JWZ was responsible for fundraising, provided administrative and material support, and supervised the study. All authors contributed to the article and approved the submitted version.
